# Shallot Aphids, *Myzus ascalonicus*, in Strawberry: Biocontrol Potential of Three Predators and Three Parasitoids

**DOI:** 10.1673/031.013.8301

**Published:** 2013-09-04

**Authors:** Annie Enkegaard, Lene Sigsgaard, Kristian Kristensen

**Affiliations:** 1 Aarhus University, Science and Technology, Department of Agroecology, Research Centre Flakkebjerg, DK-4200 Slagelse, Denmark; 2 University of Copenhagen, Faculty of Science, Department of Plant and Environmental Sciences, Thorvaldsensvej 40, DK-1871 Frederiksberg C, Denmark; 3 Aarhus University, Science and Technology, Department of Agroecology, Research Centre Foulum, Blichers Allé 20, Postboks 50, DK-8830 Tjele, Denmark

**Keywords:** *Adalia bipunctata*, *Aphelinus abdominalis*, *Aphidius colemani*, *Aphidius ervi*, *Aphidoletes aphidimyza*, *Chrysoperla carnea*

## Abstract

The parasitization capacity of 3 parasitoids and the predation capacity of 3 predators towards the shallot aphid, *Myzus ascalonicus* Doncaster (Homoptera: Aphididae), on strawberry, *Fragaria* x *ananassa* Duchesne (Rosales: Rosaceae) cv. Honeoye, were examined in laboratory experiments. In Petri dish assays, both *Aphidius colemani* Viereck (Hymenoptera: Aphidiidae) and *A. ervi* Haliday readily stung shallot aphids, with no significant difference in stinging frequency between the two species. *A. ervi* induced a significantly higher mortality (79.0 ± 7.2%) in terms of stung aphids compared with *A. colemani* (55.3 ± 4.1%); however, only a minor fraction (2.7 ± 1.8% and 7.1 ± 3.1%, respectively) of the killed aphids resulted in formation of mummies, presumably due to a physiological response to parasitism. The low percentage of mummification precludes the use of either *Aphidius* species in anything but inundative biocontrol. In similar set-ups, *Aphelinus abdominalis* (Dalman) (Hymenoptera: Aphelinidae) killed almost half (49.6 ± 5.3%) of the exposed aphids through host feeding. In addition, 23.2 ± 7.3% of non-host-fed aphids developed into mummified aphids, and 38.1 ± 13.2% of non-host-fed aphids died from other parasitoid-induced causes. However, the host feeding rate was reduced to only 1.2 ± 0.8%, and no significant parasitization mortality was observed on strawberry plants, suggesting that host plants interfered with *A. abdominalis* activity. This parasitoid does not, therefore, seem to be suited to either inoculative or inundative biocontrol of shallot aphids in strawberry. The three predators studied were the green lacewing, *Chrysoperla carnea* Steph. (Neuroptera: Chrysopidae), the two-spotted lady beetle, *Adalia bipunctata* L. (Coleoptera: Coccinellidae), and the gall midge *Aphidoletes aphidimyza* (Rondani) (Diptera: Cecidomyiidae). Third instars of all 3 predators readily preyed upon the shallot aphid in Petri dish set-ups with significant differences in daily predation (34.62 ± 3.45, 25.25 ± 3.18, and 13.34 ± 1.45, respectively). Further studies on *A. bipunctata* revealed that the larvae maintained their daily predation capacity (32.0 ± 6.3) on strawberry plants. About 60% of already ovipositing *A. bipunctata* refrained from laying any eggs on the first day after transfer to set-ups with combinations of shallot or peach-potato aphids, *Myzus persicae* (Sulzer) (Homoptera: Aphididae), and strawberry or sweet pepper leaves. The aphid species and the plant species did not, however, have a significant influence on the number of females laying eggs, the average number of eggs laid during the first day being 6.37±1.28 per female. Adult lady beetles had a significant preference for odor from controls without plants over odors from uninfested strawberry or pepper plants, but they showed no preference for either of the plant species, whether infested with aphids or not. The predation capacity of *A. bipunctata* on shallot aphids holds promise for its use in inundative biocontrol, and the results on egg laying cues suggests that inoculative biocontrol may be possible, although further studies will be needed for a complete evaluation.

## Introduction

Strawberry, *Fragaria* × *ananassa* Duchesne (Rosales: Rosaceae), is the most important high-value berry crop in Denmark for fresh consumption (Danmarks Statistik 2005; Nielsen 2006). Danish strawberry is prone to infestations with a number of pest species, e.g., the strawberry tortrix moth, *Acleris comariana*, the strawberry blossom weevil, *Anthonomus rubi*, the strawberry mite, *Phytonemus pallidus*, and the two-spotted spider mite, *Tetranychus urticae* (Lindhardt et al. 2003). In addition, new strawberry pest species known to cause serious problems in warmer climates (Karl 1983; Cross et al. 1994; Rabasse et al. 2001) are beginning to appear. One of these species is the shallot aphid, *Myzus ascalonicus* Doncaster (Homoptera: Aphididae), which can be expected to become an increasing problem in Denmark due to the global climate changes.

Aphids have not previously posed a serious problem in Danish strawberry grown in open fields or in tunnels. However, in 2008, *M. ascalonicus* infestations were observed in Denmark and Sweden in plastic-covered strawberry beds (Petersen et al. 2008). *M. ascalonicus*, known to attack strawberry in Germany (Borchardt 1958; Martin Hommes, Julius Kuehn Institute, Federal Research Centre for Cultivated Plants, Germany personal communication) and the United Kingdom (Cross et al. 1994; Anonymous 2011), can inflict serious damage on this crop due to its induction of malformed leaves, stunted growth, and shortened and distorted flower stalks (Anonymous 2011). There are indications that shallot aphids can become especially problematic in strawberry if temperatures in February and March are above normal (Hurst 1969). Winters in Denmark are expected to become milder as a consequence of global climate changes; combined with elevated temperatures during production due to the ongoing shift in strawberry cultivation methods (from open field to plastic-covered beds or in tunnels) (Daugaard 2005), this is likely to increase the importance of *M. ascalonicus* as a pest in Danish strawberry. To forestall the accompanying increase in the use of insecticides, it is important to develop biological control methods against this pest.

The literature presents only a few investigations on natural enemies of *M. ascalonicus* (Wahab 1985; Wahab and Askew 1989); however, more information exists regarding natural enemies of the strawberry aphid, *Chaetosiphon fragaefolii* (e.g., Easterbrook et al. 2006; Fitzgerald 2006). In addition, countless investigations have been made regarding the possibilities of biological control of other aphid species in various field and, especially, greenhouse crops. A number of aphidophagous natural enemies have welldocumented beneficial effects and are commercially available. However, these natural enemies are not necessarily effective against *M. ascalonicus*, which may not be included in the host or prey range of the natural enemy, may not occupy accessible spatial niches, or may display effective defense behavior (Enkegaard and Brødsgaard 2002). Consequently, this study screened a number of commercially available benificials for their predation or parasitization capacity towards *M. ascalonicus*, and further studies were undertaken with better-suited candidate species.

## Materials and Methods

The experiments were conducted at light and temperature conditions mimicking those found in spring in Danish tunnel strawberry. For an overview of experiments, see Table 1.

### Plants, insects

Onion plants, *Allium cepa* L. (Asparagales: Alliaceae) cv. Stutgarter Riesen, strawberry plants, and sweet pepper plants, *Capsicum annuum* L. (Solanales: Solanaceae) cv. Arcano, were grown separately in insect-proof netcovered cages (68 × 75 × 82 cm) in a climatecontrolled greenhouse compartment at 23 ± 1° C, 70 ± 5% RH and 16:8 L:D at Research Centre Flakkebjerg, Aarhus University, Denmark.

*M. ascalonicus* were received from the Julius Kuehn Institute, Federal Research Centre for Cultivated Plants, Braunschweig, Germany, and used to initiate a rearing. *M. ascalonicus* were reared in cages at environmental conditions as described above. Peach-potato aphids, *Myzus persicae* (Sulzer) (Homoptera: Aphididae), were obtained from a long-standing colony reared on sweet pepper and maintained at the Research Centre Flakkebjerg in similar cages and conditions.

Natural enemies (parasitoids *Aphidius colemani* Viereck (Hymenoptera: Aphidiidae), *A. ervi* Haliday, and *Aphelinus abdominalis* (Dalman) (Aphelinidae)), predators (lacewings, *Chrysoperla carnea* Steph. (Neuroptera: Chrysopidae), lady beetles, *Adalia bipunctata* L. (Coleoptera: Coccinellidae), and gall midges, *Aphidoletes aphidimyza* (Rondani) (Diptera: Cecidomyiidae)) were purchased from natural enemies producers EWH BioProduction (www.bioproduction.dk) and Borregaard Bioplant (www.bioplant.dk).

The parasitoids were supplied as mummies, which were placed according to species in open Petri dishes (diameter: 9 cm) placed in a small cage (30.5 × 22 × 15 cm) in a climate cabinet (23 ± 1° C, 70 ± 1% RH, 16:8 L:D) until emergence. The parasitoids were allowed to mate in the cage for 1 day with access to sugar water, after which females were sexed under a stereo-microscope and used for experimentation.

Gall midges were supplied as third instars and used directly in the experiments. Lacewings and lady beetles were supplied as second instars. For all experiments, except those mentioned below, larvae were reared individually in Petri dishes (diameter: 5.5 cm) to the third instar on surplus flour moth, *Ephestiakuehniella* Zeller (Lepidoptera: Pyralidae), eggs in a climate cabinet at similar conditions as above. One day prior to experimentation, predator larvae were starved for 24 hr by keeping them individually in Petri dishes in a climate cabinet in the same conditions as above.

For the behavioral experiment and the experiment with egg laying of *A. bipunctata*, the rearing of the purchased second instars continued until adulthood, after which emerged adults were kept together in groups of 15–20 individuals of similar age. Both males and females were kept together to ensure mating. *A. bipunctata* were fed on flour moth eggs, with a supplemental feeding of grain aphids, *Sitobion avenae* (F.) (Homoptera: Aphididae), every two days. For the egg-laying experiment, adult *A. bipunctata* were collected one day prior to experimentation and placed individually in small Petri dishes with the same food. The following day, females that had laid eggs during isolation were used for experimentation. For the behavioral experiments, adult *A. bipunctata* were collected 1 day prior to experimentation and placed individually in small Petri dishes without food but with access to water. After a 24-hr starvation period, the lady beetles were used for experimentation.

### Screening experiments

**Parasitoids.** A set-up used successfully in prior experiments (e.g., Enkegaard and Brødsgaard 2002) was originally intended for the screening experiments with parasitoids. In this Petri dish set-up, aphids were exposed on detached leaves to a female parasitoid for a pre-defined period depending upon the parasitoid species; they were then left for ≈1.5 weeks, after which the degree of parasitization was recorded. After several attempts, this design had to be abandoned because *M. ascalonicus* were unable to survive on detached strawberry leaves for the period needed for development of visible signs of parasitization. Instead, the following experiments were undertaken:

***Aphidius colemani* and *Aphidius ervi*.** Forrecording frequency of parasitization, Petri dishes (diameter: 9 cm) with moistened filter paper and a detached strawberry leaf (≈ 4 × 7 cm) with 25 second instar *M. ascalonicus* were each supplied to a mated female parasitoid and observed under a stereo microscope for 15 min in a climate-controlled greenhouse compartment at 18 ± 1° C. The number of aphids stung was recorded. The number of replicates was 13 and 15 for *A. colemani* and *A. ervi*, respectively. For recording actual parasitization of stung aphids, similar Petri dishes with similarly sized aphid-infested strawberry leaves were each supplied to a mated female parasitoid and observed under a stereo microscope in the same greenhouse compartment at 18 ± 1° C. For each individual parasitoid (replicate), 10 aphid nymphs observed to be stung were immediately transferred to a small, 3leafed, potted onion plant (≈ 10 cm height), which was subsequently placed in a climate cabinet (23 ± 1° C, 70 ± 1% RH, 16:8 L:D) for 1.5 weeks, after which the respective number of live, dead, and mummified aphids was recorded. The number of replicates was 13 *A. colemani* and 10 *A. ervi*. In addition, 5 onion plants, each with 10 second instar *M. ascalonicus* unexposed to parasitoids, were set up as controls.

***Aphelinus abdominalis*.** Petri dishes (diameter: 9 cm) with similarly sized aphid-infested strawberry leaves as above were each supplied to a mated female parasitoid and placed for 24 hr in a climate cabinet (18 ± 1° C, 70 ± 1% RH, 12:12 L:D), after which the numbers of dead and live aphids were recorded. For each replicate, the live aphids were, as above, subsequently transferred to a small potted onion plant placed at similar conditions and recorded in a similar fashion after 1.5 weeks. The number of replicates was 16, with 5 controls consisting of onion plants with 25 second instar *M. ascalonicus* unexposed to parasitoids.

**Predators.** For the screening experiments, a similar set-up as above was used. The Petri dishes were each supplied with a starved third instar predator and placed in a climate cabinet (18 ± 1° C, 70 ± 1% RH, 12:12 L:D). After 3 hr (*C. carnea*), 4 hr (*A. bipunctata*), or 24 hr (*A. aphidimyza*), the predator was removed and the number of consumed aphids recorded. The predation times for each predator species were defined based on the predators’ maximum daily predation capacity. The number of replicates was 25 for *C. carnea*, 25 for *A. bipunctata*, and 23 for *A. aphidimyza*. In addition, controls (n = 10) without natural enemies were used for each predator species.

### Further studies of the most suited predator and parasitoid species

***Aphelinus abdominalis*.** Experiments were conducted to examine the parasitization/host feeding potential of *A. abdominalis* on whole strawberry plants. Small cages (23 × 23 × 23 cm) with either a small 3- to 4-leafed potted strawberry plant or a small 4- to 6-leafed potted sweet pepper plant with 60 second instar *M. ascalonicus* were each supplied with 1 female *A. abdominalis* (mated, maximum 2 days old). After 24 hr in a climate cabinet (18 ± 1° C, 70 ± 1% RH, 12:12 L:D), the parasitoids were removed and the number of dead aphids recorded. For each replicate, the live aphids were subsequently transferred to a small potted onion plant (dimension as above) and placed in a climate cabinet (23 ± 1°C, 70 ± 1% RH, 16:8 L:D) for 1.5 weeks, after which the respective number of live, dead, andmummified aphids was recorded. The number of replicates was 11 for strawberry and 20 for sweet pepper. In addition, plants with 60 second instar *M. ascalonicus* unexposed to parasitoids were made as controls for each plant species (n = 10 for strawberry and n = 13 for sweet pepper).

***Adalia bipunctata* predation on whole plants.** Predation by *A. bipunctata* larvae was studied on whole plants. Similar cages as above with similarly sized aphid-infested strawberry or sweet pepper plants were each supplied with one third instar *A. bipunctata* larva (starved for 24 hr). After 24 hr in a climate cabinet (18 ± 1° C, 70 ± 1% RH, 12:12 L:D), the larvae were removed and the number of consumed aphids recorded. The number of replicates was 12 and 14 for set-ups with strawberry plants and sweet pepper plants respectively. In addition, controls without *A. bipunctata* larvae were made for each plant species (n = 6 for strawberry and n= 8 for sweet pepper).

***Adalia bipunctata* egg laying cues.** Experiments were conducted to examine if ovipositing *A. bipunctata* would obtain sufficient cues from aphid-infested strawberry to continue egg laying. Petri dishes (diameter: 9 cm) with moistened filter paper and either a strawberry leaf or a sweet pepper leaf infested with either *M. ascalonicus* or *M. persicae* (≈ 100 aphids per leaf) were each supplied with 1 adult, 6–7-day-old, female *A. bipunctata*. After 24 hr in a climate cabinet (18 ± 1° C, 70 ± 1% RH, 12:12 L:D), the females were removed and the number of eggs counted. The number of replicates was 15 for each treatment.

***Adalia bipunctata* behavior.** The reaction of adult *A. bipunctata* to odor emitted from uninfested and aphid-infested leaves wasexamined in a still-air olfactometer set-up modified after Weeks et al. (2010). The set-up consisted of a rectangular plastic box (5 × 21 × 31 cm) with the edges sealed with parafilm and with two holes (4 cm diameter, covered with insect net) at the bottom, each hole positioned at opposite ends of the box on the longest central axis and 25 cm apart. A small glass container (3 L) was placed to contain test material under each hole. The top of the container was sealed against the box with isolation tape. A single adult *A. bipunctata*, less than 1 week old and starved for 24 hr, was introduced to the center of the set-up through a small hole (1 cm diameter) in the center of the box lid. The hole was plugged, and the beetle was then observed for 5 min, during which time the time spent in each of the two halves of the box was recorded. Individuals that remained inactive for more than 2 successive minutes were discarded from the analysis. Only 2 individuals were discarded. Six experiments were conducted to examine the response of *A. bipunctata* to small 3-leafed (8–10 cm height) uninfested or aphid-infested sweet pepper or strawberry plants in the following treatment combinations: (1) uninfested strawberry plant versus empty container, (2) uninfested sweet pepper plant versus empty container, (3) uninfested strawberry plant versus uninfested sweet pepper plant, (4) aphidinfested strawberry plant versus empty container, (5) aphid-infested sweet pepper plant versus empty container, (6) aphid-infested strawberry plant versus aphid-infested sweet pepper plant. The infested material was produced by adding approximately 50 *M. persicae* (mainly nymphs) to the plants 2 days prior to experimentation. The plants were placed in a climate cabinet (23 ± 1° C, 70 ± 1% RH, 16:8 L:D) until use. The test material was placed in the containers 5 min prior to initiation of the experiment. Three batches of 5 *A. bipunctata* were tested for each treatment.

As is common practice in olfactometer studies (e.g., Souissi et al. 1998; Venzon et al. 1999), after testing each batch of insects the set-up was cleaned with 70% alcohol and left to dry for 5 min. Subsequently, odor sources were switched between the left and right side to minimize any spatial effect on choices. Each individual was tested only once. The experiments were conducted in a darkened room (23° C) with a light source positioned directly above the center of the set-up.

### Data analysis

The recorded predation was corrected for the mortality in the corresponding controls (Abbott 1925). For comparison between the three predators, the corrected predation rates were extrapolated to daily consumption (assuming predation for 12 hr of light). The number of stung, dead, and mummified aphids in the screening experiments with *A. colemani* and *A. ervi* was analyzed in one-way ANOVA with the species as fixed effects. The corrected number of dead aphids in the Petri dish screenings of *A. abdominalis, C. carnea, A. aphidimyza*, and *A. bipunctata* was analyzed in one-way ANOVA with the species as fixed effects. Because the variance depended on the species in question, a separate variance was estimated for each species. Similar analysis was applied for the whole plant experiments with *A. abdominalis* and *A. bipunctata*. The egg-laying of *A. bipunctata* was analyzed in one-way ANOVA with the four combinations of aphids and plants as fixed effects. In order to examine the behavior, the differences in time spent in the two halves of the set-up were analyzed in one-way ANOVA with pairs of treatment as fixed effects. The comparison of the two different materials in each pair was examined by testing the hypothesis that the difference was zero (this analysis is similar to a paired *t*-test except that in this analysis the variance was assumed to be the same for allpairs). All analyses were performed using procedures glm, mixed, and glimmix of SAS (SAS Institute Inc. 2008).

## Results and Discussion

Table 1 shows an overview of the experiments, and Table 2 shows a summary of the results.

### Parasitoids

**Screening of parasitization capacity for *Aphidius colemani* and *Aphidius ervi*.** Both *Aphidius* species readily stung *M. ascalonicus*, which is in contrast to a previous study undertaken with a strain of *M. ascalonicus* (obtained from Campanula, called “Campanula-strain”) with a strongly developed defensive behavior towards parasitization by both *Aphidius* species (Enkegaard and Brødsgaard 2002). The average number of stung aphids (± SE) was not significantly different (F_*1,27*_ = 0.48, *p* = 0.785) between the two parasitoid species, being 6.8 ± 2.4 and 6.1 ± 1.2 for *A. colemani* and *A. ervi* respectively. Not surprisingly, a high proportion of aphids that had been stung by either species died within 1.5 weeks. The average mortality (± SE) induced in aphids stung by *A. ervi* (79.0 ± 7.2%) was significantly higher than the mortality induced in aphids stung by *A. colemani* (55.3 ± 4.1%) (F_*1, 22*_ = 9.22, *p* = 0.0063). However, only a minor fraction (7.1 ± 3.1% and 2.7 ± 1.8% for *A. colemani* and *A. ervi* respectively) of the aphids killed after exposure to either of the parasitoids died as a result of a completed parasitization process leading to formation of mummies, with no significant difference between these values (F*_1,22_* = 1.20, *p* = 0.268).

Parasitization by the two parasitoid species leading to fully developed mummies has been reported in the literature to vary from 40 to 80% at temperatures above 20° C, depending upon the offered aphid host species, host density, and environmental conditions (e.g., Sequeira and Mackauer 1994; van Steenis 1995; Takada and Tada 2000; Zamani et al. 2007; Byeon et al. 2010). These rates, expressing mortality among all exposed aphids (i.e., not just stung aphids), are higher than the rates for mummification found in our study, indicating that the performance of *A. colemani* and *A. ervi* against *M. ascalonicus* on strawberry is far from optimal. The low degree of mummification suggests a physiological response to parasitism by *Aphidius* parasitoids in *M. ascalonicus*, as demonstrated for clones of pea aphids, *Acyrthosiphonpisum*, to parasitization by *A. ervi* (Henter and Via 1995), although the physiological mechanism described by Henter and Via (1995) served as a true defense (i.e., survival) reaction, which was not the case in our study, because the stung aphids actually died. It therefore seems that *M. ascalonicus* has the potential to form clones with resistance to parasitization based on physiological or behavioral mechanisms (Enkegaard and Brødsgaard 2002).

Even though the overall mortality rates induced in stung *M. ascalonicus* were quite high, the low degree of mummification precludes the use of either species in anything but inundative biocontrol in which no reproduction of significance can be expected. Given a choice, *A. ervi* should be preferred due to its higher mortality induction.

***Aphelinus abdominalis:* screening for parasitization capacity and parasitization on whole plants.** Contrary to the 2 *Aphidius* species, *A. abdominalis* uses its hosts for host feeding (Stary 1988; Haardt 1990), as evidenced in the present experiment with almost half (49.6 ± 5.3%) of the aphids being killed within 24 hr after exposure to the parasitoids.

In addition, *A. abdominalis* inflicted additional mortality to *M. ascalonicus*, with 23.2 ± 7.3% of the non-host-fed aphids developing into mummified aphids, and 38.1 ± 13.2% of the non-host-fed aphids dying from parasitoidinduced causes; this was presumably a result of parasitizations that did not develop to the mummified stage.

The results found for *A. abdominalis* are in contrast to the study by Enkegaard and Brødsgaard (2002) in which the defensive behavior of the Campanula strain of *M. ascalonicus* was obstructive to parasitization by *A. abdominalis*. On the other hand, our findings agree with the study of Wahab (1985) in which dissections of *M. ascalonicus* 3 days after exposure to *A. abdominalis* at 18° C revealed that 40–70% of the aphids contained parasitoid eggs or larvae. The method used by Wahab (1985) allowed for neither determination of mortality due to host-feeding nor determination of the proportion of parasitized aphids that would eventually have mummified. However, in view of reports on successful aphid biocontrol with *A. abdominalis* (e.g., Hurni and Stadler 1993; Colombo and Fasce 1994; Blumel and Hausdorf 1996), it seems probable that the majority of the parasitized aphids in the study of Wahab (1985) would have mummified. The lower degree of mummification found in our study was therefore likely a reflection of the presumed physiological mechanism mentioned above for the *Aphidius* species.

*Aphelinus abdominalis* and *A. ervi* inflicted the same overall mortality on *M. ascalonicus* (about 80%), although the mortality inflicted by the latter was based only on stung aphids. The mortality to be expected among a group of exposed *M. ascalonicus* would thus be higher for *A. abdominalis*, which, combined with its ability to reproduce on this aphid species, at first would seem to make it a better candidate for biological control with the potential to be used as an inoculative agent. However, the results from the experiment on realization of the potential of *A. abdominalis* on whole plants showed otherwise.

On whole plants, the host feeding mortality inflicted by *A. abdominalis* on *M. ascalonicus* was significantly influenced by the host plant species (*p* < 0.0001, F_1,29_ = 38.45), with a corrected host feeding mortality (± SE) of 32.1 ± 5.3% on sweet pepper and only 1.2 ± 0.8% on strawberry. Compared with the relatively high host feeding mortality observed on strawberry leaves, the latter result suggests that qualities pertaining to whole strawberry plants were obstructive to *A. abdominalis* activity. Contrary to the results obtained in the screening experiment, no significant parasitization mortality was observed in the whole plant set-up with either of the host plants (strawberry: ñ = 0.73, F_1,20_ = 0.12; sweet pepper: *p* = 0.83, F_1,32_ = 0.05). Thus, the obstructive factors pertained to both host-feeding and parasitization activities. It may be speculated that the trichomes of strawberry plants constituted an obstacle for parasitoid search, which primarily is based on walking since this species seldom flies (Biobest 2011). Another possibility is that volatile signals emitted from shallot aphid-infested strawberry plants were in some way discouraging to the parasitoids (Mölck et al. 1999; Mölck and Wyss 2003). On sweet pepper, the occurrence of host feeding but the lack of parasitization was surprising because an obstructive influence from either plant structure (other than trichomes because sweet pepper is void of trichomes (Madadi et al. 2007)) or pest-plant volatiles would have been expected to affect both behavioral elements more or less equally.

Even though *A. abdominalis* may have some potential to control shallot aphids on sweet pepper, the same potential is not likely on strawberry when used in an inundative fashion.

### Predators

**Screening of predation capacity.** Third instar larvae of all 3 predators readily preyed upon *M*. ascalonicus, with a significant difference (F*_2, 70_* = 19.34, *p* < 0.0001) in daily predation between predator species. The average daily predation rates (± SE) of larvae of *A. aphidimyza, A. bipunctata*, and *C. carnea* were 13.34 ± 1.45, 25.25 ± 3.18, and 34.62 ± 3.45, respectively. These results are within the range of predation rates reported on various aphid species for *A. bipunctata* (Timms et al. 2008; Jalali et al. 2009a), *C. carnea* (Athhan et al. 2004; Montoya-Alvarez et al. 2010), and A *aphidimyza* (Morse and Croft 1987; Kulp et al. 1989; Harizanova and Ekbom 1997). *C. carnea* larvae were more voracious than larvae of both *A. bipunctata* (t_70_ = -2.00, *p* = 0.0498) and *A. aphidimyza* (t_70_ = -5.68, *p* < 0.0001), and *A. bipunctata* larvae were more voracious than *A. aphidimyza* larvae (t_70_ = 3.40,*p* = 0.0011).

**Predation by *Adalia bipunctata* on whole plants.** Third instar *A. bipunctata* had significantly higher predation (F*_1,24_* = 14.17, *p* < 0.001) on *M. ascalonicus* on sweet pepper (average daily corrected number eaten (± SE) = 56.3 ±1.7) than on strawberry (32.0 ± 6.3), with the latter predation rate being in accordance with that observed in the screening experiment. Contrary to the situation with *A. abdominalis*, the change in experimental dimensions did not seem to be obstructive to the activity of the *A. bipunctata* larvae. However, the difference in predation on the 2 host plant species showed that larvae were influenced by prey-plant characteristics (Timms et al. 2008;Alhmedi et al. 2010), perhaps due to the structure or texture of the plants (Banks 1957; Shah 1982; Carter et al. 1984).

**Egg laying cues for *Adalia bipunctata*.** A high proportion of the already ovipositing *A. bipunctata* refrained from laying any eggs during the 24 hr experimental period (strawberry-*M. ascalonicus:* 46.6%, strawberry-*M. persicae:* 53.3%, sweet pepper-*M. ascalonicus:* 66.7%, sweet pepper-*M. persicae:* 66.7%). The number of females refraining from egg-laying was, however, not affected by the species of the aphid and the species of the plant (χ^2^ = 0.038, df = 1,*p* = 0.845). The results are in accordance with the study by Hemptinne et al. (2000), which demonstrated that female *A. bipunctata* use cues other than those associated with aphids or plants in their oviposition site selection.

The average number (± SE) of eggs laid per day by *A. bipunctata* was similar, irrespective of aphid and plant species (F*1,59* = 1.07, *p* = 0.369), with the following values attained in each plant-aphid system: 8.67 ± 3.08 eggs (strawberry-*M. ascalonicus*), 8.07 ± 2.91 eggs (strawberry-*M. persicae*), 5.93 ± 2.59 eggs (sweet pepper-*M. ascalonicus)*, and 2.8 ± 1.24 eggs (sweet pepper- *M. persicae)*. Since the *A. bipunctata* in this experiment were only allowed a short egg-laying period with the new prey, the average daily egg laying across all treatments (6.37 ± 1.28) was, not surprisingly, lower than reported in other studies where egg production was observed for longer periods (16–20 eggs) (Hamalainen et al. 1975; Ware et al. 2008; Jalali et al. 2009b). Further studies will be needed to examine if the proportion of egg layers as well as the egg-laying capacity of *A. bipunctata* would increase after longer exposure to *M. ascalonicus* on strawberry.

**Behavior of *Adalia bipunctata*.** The time spent by adult *A. bipunctata* over the different odor sources of uninfested and aphid-infested plant material is shown in Table 3. *A. bipunctata* had a significant preference for clean air from the empty container over odors from uninfested strawberry (trial 1), and there was a tendency for a similar preference for clean air over uninfested sweet pepper (trial 2). This apparent repellency of both plant species is in accordance with the findings of Timms et al. (2008), who found a significant negative olfactory response of adult *A. bipunctata* to uninfested Norway spruce compared to controls without plants. However, the results contradict previous observations that adult *A. bipunctata* are not attracted to volatiles from uninfested broad beans, rape, or mustard (Francis et al. 2004), or to volatiles from uninfested broad bean or Nasturtium plants (Raymond et al. 2000). Avoidance or attraction of *A. bipunctata* to plant volatiles apparently depends upon the plant species in question. When *A. bipunctata* was given a choice between both uninfested plant species (trial 3), neither was preferred, as was also the case when they were given a choice between both infested plant species (trial 6). However, the presence of aphids made both plant species more attractive since *A. bipunctata* no longer displayed a significant preference for clean air (comparison of trials 1 and 4 and of trials 2 and 5). Still, only trials 1 and 4 showed a tendency to being significantly different (t = -1.86, *p* = 0.066), whereas this was not the case for trials 2 and 5 (t = -1.35, *p* = 0.189). This result is in accordance with the findings of Raymond et al. (2000), who observed a tendency for *A. bipunctata* with previous feeding experience to show an olfactory preference for broad bean or Nasturtium plants infested with black bean aphids, *Aphis faba*, over uninfested plants.

Although *A. bipunctata* seems to find strawberry an inferior host plant compared to sweet pepper, its realized predation capacity on *M. ascalonicus* on strawberry indicates a potential in inundative biocontrol. In addition, the results on eg- laying cues suggest that *A. bipunctata* may be used in inoculative biocontrol strategies, provided that adults developing from released larvae will remain in the culture to initiate further generations. Even though *A. bipunctata* is apparently repelled by uninfested strawberry, the extent of this reaction in the presence of aphids suggests that this may indeed be the case. It might consequently be expected that adult *A. bipunctata* developing from released larvae will remain in aphidinfested strawberries. It would be valuable to investigate if short-term learning processes, as has been documented for the seven-spotted lady beetle, *Coccinella septempunctata* (Glinwood et al. 2011), in adult *A. bipunctata* developing from larvae released in a strawberry culture may increase their tendency to remain in the crop.

### Conclusion

This study has demonstrated that the reproductive success of *A. colemani, A. ervi*, and *A. abdominalis* is low on *M. ascalonicus* compared to the abilities of these parasitoids on other aphid species. The low reproductive success is not a result of behavioral defense reactions towards the parasitoids. Instead, *M. ascalonicus* seemingly responds physiologically to the internal presence of parasitoid eggs or larvae, although this reaction does not serve as an effective defense mechanism as demonstrated in other aphid species (Henter and Via 1995), since the aphids themselves die. The very low reproductive success of the two *Aphidius* species precludes their use in biocontrol against *M. ascalonicus* in strawberry except for purely inundative releases. In addition, the reduced host- feeding and reproductive capacity of *A. abdominalis* on whole strawberry plants compared to the initial screening experiments likewise precludes the use of this species even as an inundative agent. Whether other parasitoid species than the 3 species examined here will be able to parasitize *M. ascalonicus* on whole strawberry plants remains to be seen. It likewise remains to be seen if other *M. ascalonicus* populations will exhibit the same resilience to parasitization as seen here.

All predator larvae readily preyed upon *M. ascalonicus* on strawberry, with *C. carnea* larvae being the most voracious. Although *A. bipunctata* seems promising as a biocontrol agent, as judged from its predation capacity and its ability (at least for about half of the females) to lay eggs on *M. ascalonicus*infested strawberries, further studies are needed to examine its departing tendencies in strawberry. Furthermore, studies are needed on realized fertility, development, and survival for individuals feeding continuously on *M. ascalonicus*. At the same time, it would be interesting to further examine the two other predators, especially *C. carnea*, to clarify the realization of their predation potential under more realistic conditions.

**Table 1.**
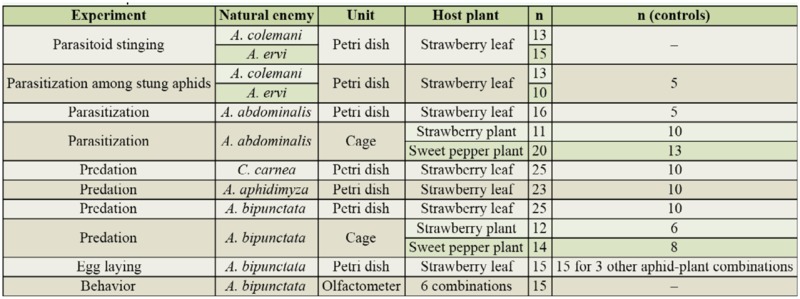
Overview of experiments.

**Table 2.**
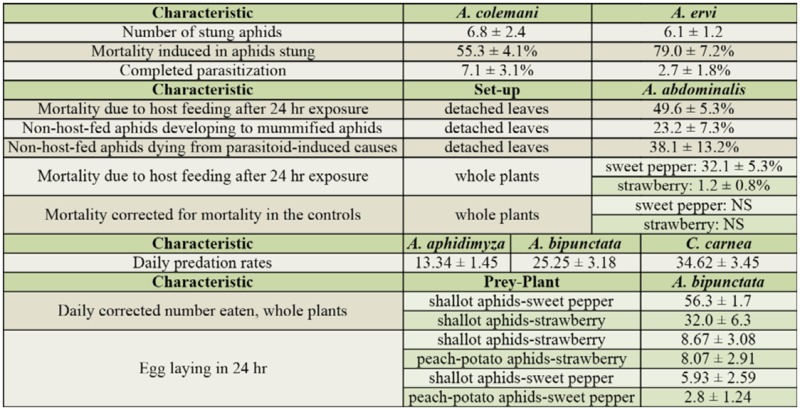
Summary of the average (± SE) values for the different biological characteristics examined for the 3 parasitoids (*Aphidius colemani, Aphidius ervi, Aphelinus abdominalis*) and 3 predators (*Aphidoletes aphidimyza, Chrysoperla carnea, Adalia bipunctata*) with shallot aphids, Myzus *ascalonicus*, as host/prey. NS: mortality was not significantly different from the controls.

**Table 3.**
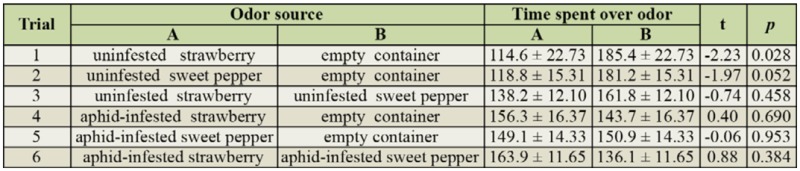
Average time (± SE) (in sec) over the different odor sources in the 6 treatment combinations, as well as the test statistics for differences in preference between the two odor sources.
